# Ultradian rhythms in accelerometric and autonomic data vary based on seizure occurrence in paediatric epilepsy patients

**DOI:** 10.1093/braincomms/fcae034

**Published:** 2024-02-12

**Authors:** Solveig Vieluf, Sarah Cantley, Vaishnav Krishnan, Tobias Loddenkemper

**Affiliations:** Division of Epilepsy and Clinical Neurophysiology, Boston Children’s Hospital, Harvard Medical School, Boston, MA 02115, USA; Department of Medicine I, LMU University Hospital, LMU Munich, 81377 Munich, Germany; Division of Epilepsy and Clinical Neurophysiology, Boston Children’s Hospital, Harvard Medical School, Boston, MA 02115, USA; Departments of Neurology, Neuroscience and Psychiatry & Behavioral Sciences, Baylor College of Medicine, Houston, TX 77030, USA; Division of Epilepsy and Clinical Neurophysiology, Boston Children’s Hospital, Harvard Medical School, Boston, MA 02115, USA

**Keywords:** cyclic patterns, autonomic activity, accelerometry, epilepsy, paediatric patients

## Abstract

Ultradian rhythms are physiological oscillations that resonate with period lengths shorter than 24 hours. This study examined the expression of ultradian rhythms in patients with epilepsy, a disease defined by an enduring seizure risk that may vary cyclically. Using a wearable device, we recorded heart rate, body temperature, electrodermal activity and limb accelerometry in patients admitted to the paediatric epilepsy monitoring unit. In our case–control design, we included recordings from 29 patients with tonic–clonic seizures and 29 non-seizing controls. We spectrally decomposed each signal to identify cycle lengths of interest and compared average spectral power- and period-related markers between groups. Additionally, we related seizure occurrence to the phase of ultradian rhythm in patients with recorded seizures. We observed prominent 2- and 4-hour-long ultradian rhythms of accelerometry, as well as 4-hour-long oscillations in heart rate. Patients with seizures displayed a higher peak power in the 2-hour accelerometry rhythm (U = 287, *P* = 0.038) and a period-lengthened 4-hour heart rate rhythm (U = 291.5, *P* = 0.037). Those that seized also displayed greater mean rhythmic electrodermal activity (U = 261; *P* = 0.013). Most seizures occurred during the falling-to-trough quarter phase of accelerometric rhythms (13 out of 27, χ^2^ = 8.41, *P* = 0.038). Fluctuations in seizure risk or the occurrence of seizures may interrelate with ultradian rhythms of movement and autonomic function. Longitudinal assessments of ultradian patterns in larger patient samples may enable us to understand how such rhythms may improve the temporal precision of seizure forecasting models.

## Teaser

Ultradian rhythms are biological oscillations of activity and physiological signals and may relate to clinically meaningful variations in the severity of chronic illness. Vieluf *et al*. examine the associations between seizure occurrence and ultradian rhythm morphology in a paediatric epilepsy cohort.

## Graphical Abstract

**Graphical Abstract:**
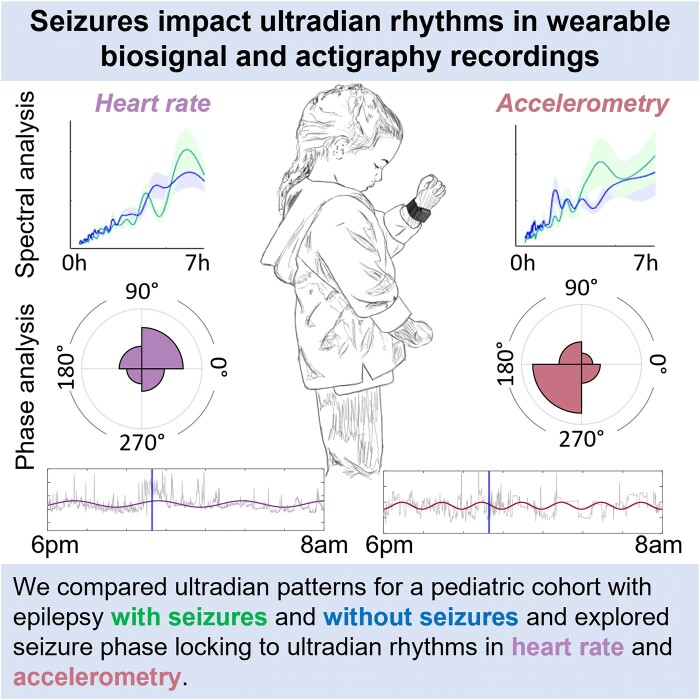

## Introduction

For people with epilepsy, the unpredictability of seizure occurrence imposes a unique set of safety concerns, including the risk of injury and medical complications. Patients report that the unpredictability of seizures causes the greatest burden on their daily life routines.^1^ Reliable seizure detection and prediction systems could help patients gain a sense of control by overcoming the uncertainty of impending seizures.^2^ With the aid of large-scale seizure diary studies^3^ and chronic ambulatory electrocorticography,^4-7^ we have gained a greater appreciation of the underlying rhythmicity of seizure occurrence in people with epilepsy. A deeper understanding of these seizure occurrences and seizure risk patterns, as well as the potential association of seizures with rhythms in physiological data, offers entry points to improving strategies to forecast and predict seizure occurrence.^8-10^

The recurrence of epileptic phenomena and events in often rhythmic intervals is one of the main characteristics of the disease.^11^ Rhythmic seizure patterns, and associated variations in seizure risk, can be observed over multiple time scales. Circadian and multiday/infradian patterns of seizure occurrence have thus far received the greatest emphasis.^12^ These studies show that interictal epileptiform activity follows a strong 24-hour periodicity and seizure occurrence relates to circadian, sleep–wake-related patterns and infradian rhythmicity.^13,14^ Beyond cycles of interictal epileptiform discharge rate, variations in seizure risk and occurrence have been associated with changes in physiological rhythms. Specifically, cardiac and electrodermal activity differ between patients with and without seizures.^15-17^ Additionally, the phases of the cyclic patterns (e.g. daily and multiday patterns of heart rate activity, as well as electrodermal activity, accelerometry and temperature) relate to the timing of seizure occurrence,^15,18,19^ which is consistent with EEG findings.

Complementary to circadian or infradian rhythms, many organisms also display ultradian rhythms, with period lengths < 24 hours, typically lasting between 1 and 12 hours.^20,21^ Compared with circadian and multiday rhythms, ultradian rhythms have received considerably less research focus. Ultradian rhythms are hypothesized to function as frequency standards for the circadian rhythm and take on a more dominant role in extreme environments (e.g. severely shortened days in winter).^22^ Such rhythms in spontaneous movement predominate in preterm and term human neonates.^23^ Ultradian rhythms have been most extensively studied in laboratory rodent subjects^24,25^ where their occurrence can be more clearly appraised by rendering subjects circadian-incompetent (achieved through a combination of continuous light or dark exposure, ‘and’ genetic deletions of circadian genes or suprachiasmatic ablation). In mice, 4- to 5-hour-long ultradian rhythms of rest and activity are driven by a tuneable midbrain dopaminergic oscillator.^26^ Similar murine rhythms in body temperature have been shown to vary with aging and in models of Huntington’s disease.^27^ The pulsatile release of several endocrine hormones also occurs with ultradian period lengths, including growth hormone,^28^ corticotropin-releasing hormone^29^ and corticosteroids.^30-32^ Ultradian rhythms contain information about shifts or disruptions of rest-activity patterns, which are common triggers for patients with paroxysmal disorders such as migraine headaches or epileptic seizures.^33-35^ In the context of epilepsy research, knowledge of cyclical patterns and the occurrence of symptoms, such as seizures, may provide an opportunity to evaluate and improve care for chronic diseases related to cyclical patterns.

In this exploratory study, we examined patterns of ultradian rhythmicity in human subjects with epilepsy via an established wearable device^36,37^ designed to simultaneously measure activity (wrist-accelerometry) and three distinct measures of autonomic balance (heart rate, body temperature and electrodermal activity). We approached these measures as physiological readouts of brain networks that subserve arousal and autonomic function, and which are intimately and bidirectionally linked with activity within distinct hypersynchronous networks that mediate fluctuations in seizure risk. We, therefore, hypothesized that variations in seizure occurrence would be associated with stereotyped distortions of ultradian rhythm expression, assessed using conventional spectral decomposition techniques.

## Materials and methods

### Ethics approval and patient consent

We designed the study protocol in line with all applicable government regulations and the Declaration of Helsinki. Boston Children’s Hospital Institutional Review Board approved the study (IRB-P00001945). Before enrolment, all participants and/or their guardians gave written informed consent.

### Patient inclusion, exclusion and data collection for clinical cohort description

**Figure 1:**
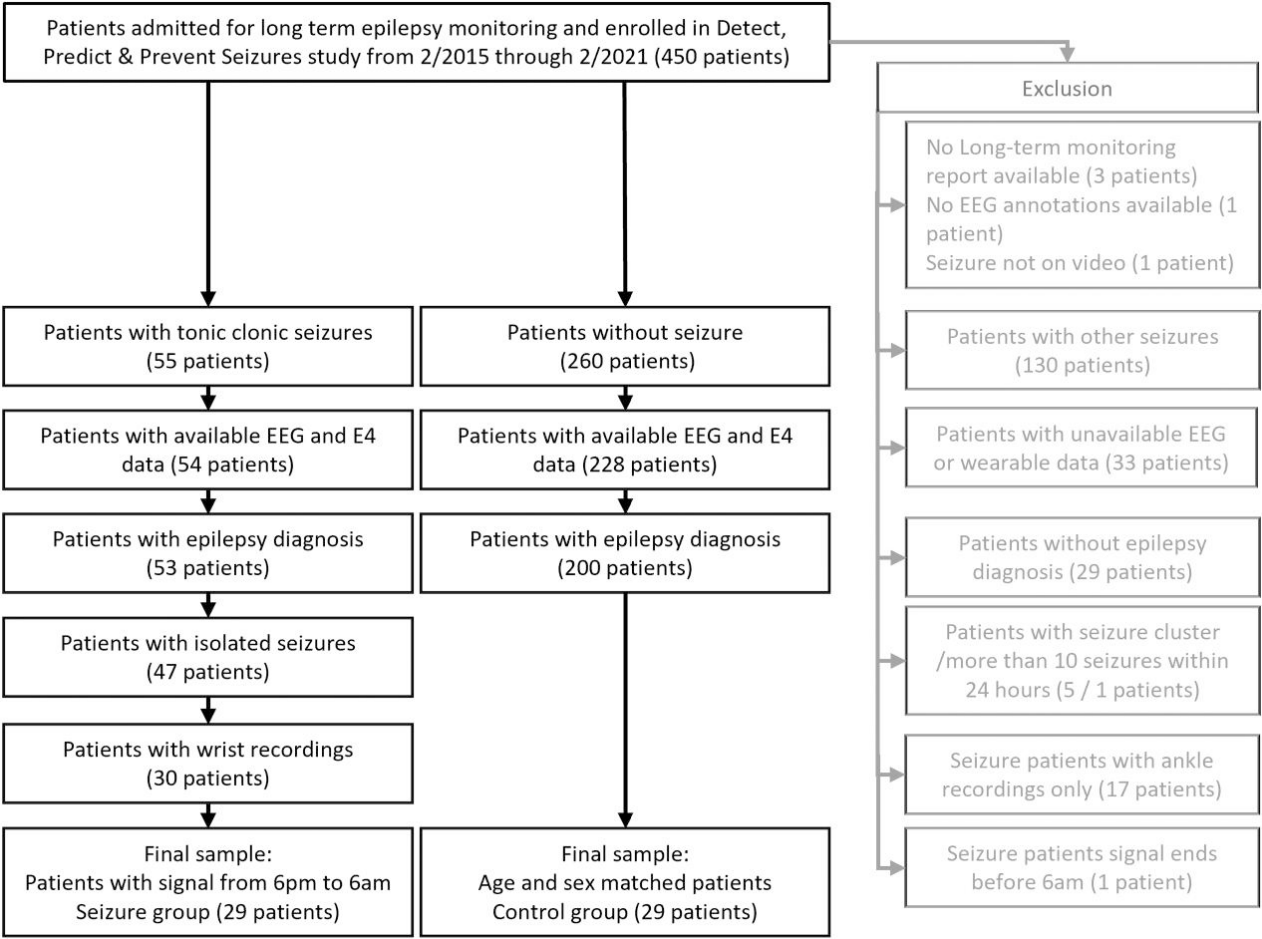
**Inclusion diagram.** Summary of the selection of 58 out of 450 patients based on the study-specific inclusion and exclusion criteria.

We collected data as part of the ‘Detect, Predict & Prevent Seizures’ study conducted at Boston Children’s Hospital.^16,38-40^ Wearable recordings (E4, Empatica Inc., Milan, Italy) were collected from patients admitted for video-EEG monitoring at the epilepsy monitoring unit (EMU) between February 2015 and February 2021. Our cohort included patients that underwent presurgical or phase I workup. For this retrospective and exploratory analysis, we performed a case–control matching. We first identified recordings from patients (i) that displayed definite tonic–clonic seizures (TCSs, of focal or generalized onset) during their EMU stay; (ii) had a diagnosis of epilepsy and a full complement of clinical data; (iii) who did not display seizure clusters (defined as ≥4 seizures in 15 minutes^41^); and (iv) with complete simultaneous EEG and wearable recordings. TCS patients were included if they had additional non-TCS (Supplementary Table 1). Next, we identified an age-and-sex-matched control group of subjects (1:1) with epilepsy who did not experience a seizure during their EMU stay but who otherwise fulfilled the same inclusion criteria (Fig. 1). If multiple wrist recordings were available per patient, we included the earliest recording, favouring the left, often non-dominant hand. We excluded ankle recordings. We tallied age at admission, age at first seizure, sex, aetiology of epilepsy, MRI finding, interictal EEG findings, type and the number of antiseizure medications (ASMs). Study data were collected and managed using REDCap electronic data capture tools hosted at Boston Children’s Hospital.^42,43^

### Data recording and processing

From E4 recordings, we analysed accelerometry (ACC, sampled at 32 Hz), heart rate data (HR, 1 Hz), electrodermal activity (EDA, 4 Hz) and peripheral body temperature data (TEMP, 4 Hz). We used MATLAB (R2019b, The MathWorks Inc., Natick, MA, USA) for signal processing. Actual wearable start- and end-times varied somewhat between patients, as the timing of study enrolment and device removal were designed to avoid any disruptions to standard clinical procedures in the EMU. For these reasons, we focused our analysis on the window between 6 pm and 8 am and included the complete 14 hours of recording from each patient. Based on enrolment times, this time window allowed for maximal dataset size of complete recordings in the TCS group, with concomitant video EEG recordings to confirm seizures. Continuous three-dimensional acceleration data were converted into a single acceleration vector by calculating the root-sum-of-squares. For each signal, i.e. ACC, HR, EDA and TEMP, we calculated the power spectral density by plotting Lomb–Scargle Periodograms (MATLAB, ‘plomb’), sampling a set of 260 frequencies with period lengths from 0.5 to 7 hours in equally spaced 0.025-hour increments. For an exploratory qualitative analysis of group differences, we plotted mean curves of time series data. By visual inspection, we selected markers of interest from visualizations and ran statistical comparisons.

To understand the relationships between the timing of TCS occurrence and the phase of ultradian rhythm, we individually regressed wearable time series data to sinewaves with a patient-specific ultradian rhythm period. We examined the phase of TCS occurrence and compared if the phase is equal for multiple seizures for patients with more than one seizure.

### Statistical analysis

Statistical analysis was performed with SPSS version 23 (IBM Corp., Armonk, New York, USA). For demographic and clinical variables, as well as warble mean values, we calculated Pearson Chi-square tests for categorical and Mann–Whitney U-tests for continuous variables to compare groups (see Table 1). To compare differences in ultradian rhythms between seizure and control patients as two independent samples, we calculated the non-parametric Mann–Whitney U-test for the outcome variables ACC 2-hour and 4-hour peak power, HR 4-hour peak power and peak period length, EDA mean power and TEMP 3-hour peak power. We report U and *P*-values. To test for differences in seizure occurrence between sinewave quarters of modelled periodic activity in ACC and HR cycles, we calculated Chi-square tests and report χ^2^ and *P*-values. We set the significance level to *P* < 0.05.

**Table 1:** Group-wise demographic and clinical characteristics of all patients included in the analysis, as well as descriptive values of wearable measures for all included recordings

Demographics variable	Answer categories	Reported in	All patients		Seizure group		Control group		*P*-value
Sex	Male	*N*, %	34	58.62	17	58.62	17	58.62	1.00
	Female	*N*, %	24	41.38	12	41.38	12	41.38	
Ethnicity	Hispanic or Latino	*N*, %	6	10.34	3	10.34	3	10.34	0.31
	Not Hispanic or Latino	*N*, %	44	75.86	24	82.76	20	68.97	
	Not reported	*N*, %	8	13.79	2	6.90	6	20.69	
Race	White	*N*, %	41	70.69	20	68.97	21	72.41	0.08
	Black or African American	*N*, %	6	10.34	1	3.45	5	17.24	
	Unknown	*N*, %	11	18.97	8	27.59	3	10.34	
Age at enrolment	In years	Median, IQR	13.45	5.25	13.75	5.05	13.5	5.18	0.89
Age at first seizure	In years	Median, IQR	6.5	9.83	8.68	9.33	4.25	7.71	0.05
Seizure frequency	Estimated per 30 days	Median, IQR	8.58	36.5	6.44	29.15	8.58	77.265	0.45
Aetiology of epilepsy	Structural	*N*, %	23	39.66	12	41.38	11	37.93	0.35
	Genetic	*N*, %	1	1.72	1	3.45	0	0.00	
	Infectious	*N*, %	1	1.72	1	3.45	0	0.00	
	Metabolic	*N*, %	1	1.72	0	0.00	1	3.45	
	Immune	*N*, %	2	3.45	2	6.90	0	0.00	
	Unknown	*N*, %	30	51.72	13	44.83	17	58.62	
Interictal EEG^a^	Normal	*N*, %	7	12.07	1	3.45	6	20.69	0.04
	Abnormal	*N*, %	51	87.93	28	96.55	23	79.31	
MRI findings	Normal	*N*, %	10	17.24	5	17.24	5	17.24	< 0.01
	Abnormal	*N*, %	20	34.48	16	55.17	4	13.79	
	Not available	*N*, %	28	48.28	8	27.59	20	68.97	
# of daily ASMs	Number	Median, IQR	2	2	3	2	2	1	0.04
Wristband location	Left wrist	*N*, %	37	63.79	21	72.41	16	55.17	0.17
	Right wrist	*N*, %	21	36.21	8	27.59	13	44.83	
Accelerometry	S. methods	Median, IQR	63.87	1.07	63.39	1.19	64.15	0.8	0.08
Heart rate	bpm	Median, IQR	78.86	15.54	81.44	18.32	77.78	13.94	0.32
Electrodermal activity	µS	Median, IQR	0.56	2.26	0.53	2.10	0.57	2.43	0.80
Temperature at wrist	°C	Median, IQR	34.91	1.74	34.94	1.75	34.76	2.06	0.70

We calculated Pearson Chi-square tests for categorical and Mann–Whitney U-tests for continuous variables to compare groups. EEG, electroencephalogram; ASM, antiseizure medication. ^a^Patients with interictal abnormalities may have had more than one abnormality.

## Results

### Patients

We included 29 patients with TCSs and 29 matched control patients (Fig. 1), aged 8 to 25 years, with 12 female and 17 male patients in each group (Table 1). Groups were comparable in age at enrolment, sex, ethnicity, race, seizure frequency estimated over 30 days, and epilepsy aetiology. Compared to controls, patients in the TCS group were older when they experienced their first seizure, had more interictal EEG and MRI abnormalities and were prescribed more ASMs at the time of EMU admission. Mean raw measures of autonomic activity and accelerometry across the recording did not differ between groups (Table 1 and Supplementary Table 2).

### Ultradian rhythms

**Figure 2:**
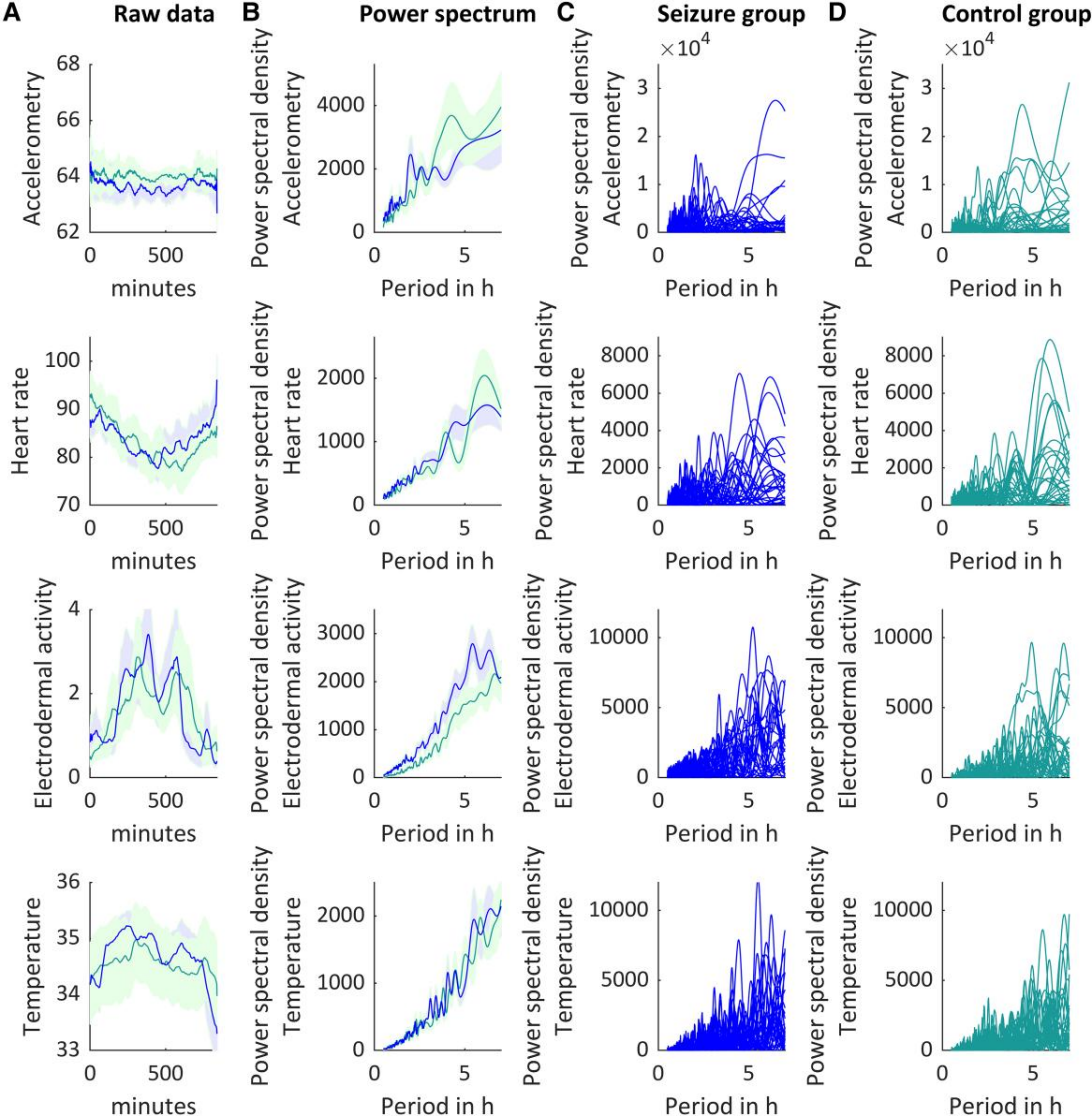
**Raw data and power spectral densities.** Illustration of 14 hours of accelerometry (1st row), heart rate (2nd row), electrodermal activity (3rd row) and peripheral body temperature (4th row) recordings, smoothed over 40 data points (average filter). Means and standard error of raw data (**A**, averaged per minute) and power spectral density (**B**) for 29 control patients (green) and 29 seizure patients (blue). (**C**) Twenty-nine individual seizure patients’ ultradian rhythms. (**D**) Twenty-nine individual control patients’ ultradian rhythms.

**Figure 3:**
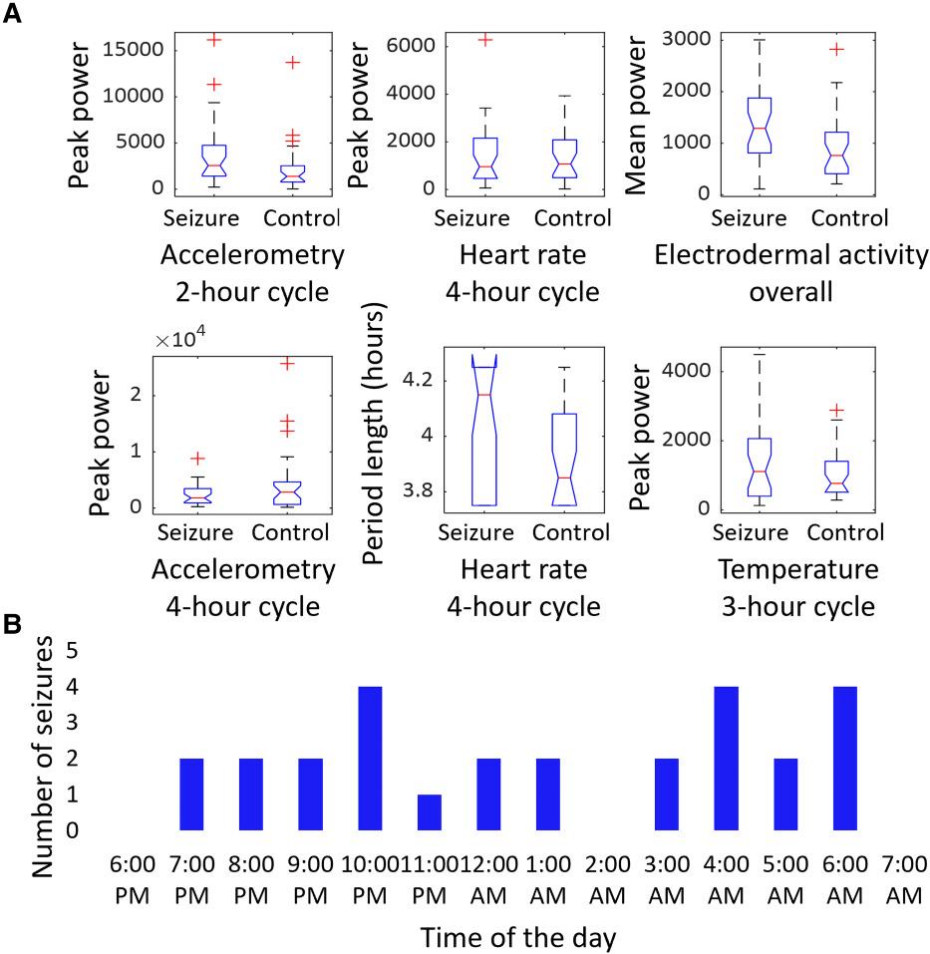
**Statistics. A** shows boxplots of markers of interest (accelerometry 2-hour, U = 287, *P* = 0.038, and 4-hour peak power, U = 473, *P* = 0.414, heart rate 4-hour peak power, U = 412, *P* = 0.895, and peak period length, U = 291.5, *P* = 0.037, electrodermal activity mean power, U = 261; *P* = 0.013, and peripheral body temperature 3-hour peak power, U = 383, *P* = 0.560) per group. Each group consisted of 29 patients and group differences were tested by Mann–Whitney U-test. The red line indicates the mean, the *bottom* and *top* edges of the box indicate the 25th and 75th percentiles and the whiskers show the range for the most extreme data points that are not considered outliers, as indicated by red plus signs. **B** depicts the distribution of the 27 tonic–clonic seizures that were recorded during the analysed segments split by each hour of the 14-hour recording period.

Spectral analyses revealed a set of prominent peaks of ultradian rhythmicity (Fig. 2). We identified the following markers of interest: ACC 2-hour peak power (range: 1.75–2.25 hours) and 4-hour peak power (range: 3.75–4.25 hours), HR 4-hour peak power and peak period length (range: 3.75–4.25 hours), EDA mean power (range: 0.5–7 hours) and TEMP 3-hour peak power (range: 2.75–3.25 hours) (Fig. 3A). For accelerometry data, the peak power ∼2 hours was higher for TCS than for control patients (U = 287, *P* = 0.038), while peak power at 4 hours did not differ between groups (U = 473, *P* = 0.414). The peak cycle length associated with the 4 hours peak in heart rate data was higher for TCS than for control patients (U = 291.5, *P* = 0.037). The mean spectral density power of EDA was lower for controls than for TCS (U = 261; *P* = 0.013). The peak power of ∼3 hours in temperature recordings did not differ between groups (U = 383, *P* = 0.560).

### Seizure occurrence related to 2-hour cycles in accelerometry and 4-hour cycles in heart rate

**Figure 4:**
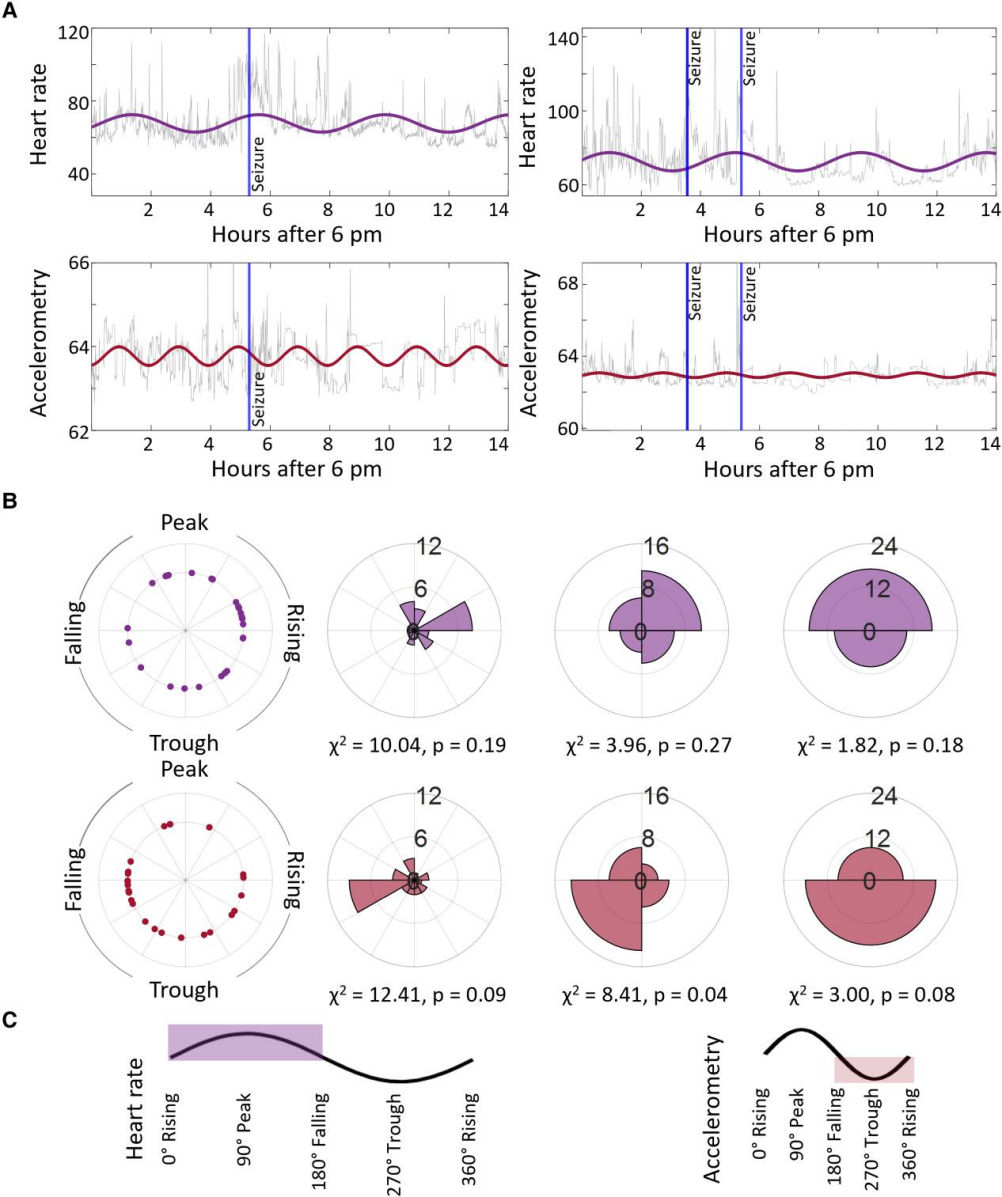
**Seizures and cycles. A** illustrates two patients’ sinewave of 4-hour range max period length fitted for heart rate (grey = raw data, purple = fitted data) and 2-hour range max period length fitted for accelerometry (grey = raw data, red = fitted data) data. For visualization only, data were averaged per minute. Blue vertical lines illustrate the electrographic seizure onset. **B** shows cyclic phases for each of the 27 seizures (left), counts added up for 12 bins (*middle*-left), counts added up for 4 bins (*middle*-right) and counts added up for 2 bins (right). **C** depicts the sinewaves’ phases.

Twenty out of 29 patients in the TCS group displayed a total of 27 TCSs during the recording (Fig. 3B, Table 2; Fig. 4A for individual examples). The average seizure duration was 88.63 ± 35.18 s. Of those seizures, 1 presented with the generalized onset and 26 with focal onsets evolving to bilateral TCSs with awareness. All 27 seizures had onsets with motor symptoms. Fourteen seizures emerged out of non rapid eye movement sleep, and the rest occurred during wakefulness. See Table 2 for a more detailed TCS description and Supplementary Table 1 for information on additional non-TCSs. Lastly, we employed phase binning to understand how seizure occurrence fluctuated with the phase of these rhythms. With two-phase binning, most seizures (17/27) occurred during the peak phase of 4-hour-long heart rate rhythms and during the trough phase of 2-hour-long rhythms of accelerometry (18/27), but this fell short of statistical significance (Fig. 4B). With four-phase binning of accelerometry rhythms, a majority of seizures (13/27) occurred in the falling-to-trough phase (χ^2^ = 8.41, *P* = 0.038).

**Table 2:** Tonic–clonic seizure semiology for 44 seizures of 29 patients

Timeframe	Total		Before 6 pm		6 pm–8 am		After 8 am	
ILAE 2017 seizure semiology	GTCS	FBTCS	GTCS	FBTCS	GTCS	FBTCS	GTCS	FBTCS
Number of patients, *N*^a^	4	25	2	5	1	19	2	8
Number of seizures, n^b^	5	39	2	5	1	26	2	8
Wake, *N* (n)	3 (4)	17 (21)	1 (1)	5 (5)	1 (1)	10 (12)	2 (2)	4 (4)
Sleep, *N* (n)	1 (1)	11 (18)	1 (1)	0	0	11 (14)	0	4 (4)
Stage I	0	3 (4)	0	0	0	3 (3)	0	1 (1)
Stage II	1 (1)	6 (10)	1 (1)	0	0	5 (7)	0	3 (3)
Stage III	0	3 (4)	0	0	0	3 (4)	0	0
REM	0	0	0	0	0	0	0	0
PGES present after seizure, *N* (n)	2 (2)	7 (13)	2 (2)	2 (2)	0 (0)	5 (8)	0	3 (3)
Duration in seconds, Median (IQR)	22 (24.5)	37 (15.5)	27 (5)	38 (15.5)	0 (0)	40 (13)	0 (0)	34 (23)

ILAE, International League Against Epilepsy; GTCS, generalized tonic–clonic seizure; FBTCS, focal to bilateral tonic–clonic seizure; REM, rapid eye movement; PGES, postictal generalized electroencephalography suppression. ^a^*N* represents the number of patients; some patients are represented in more than one group. ^b^n represents the number of seizures.

## Discussion

### Summary

In this exploratory data analysis, we examined wearable data recorded from paediatric patients with epilepsy during their stay at the EMU to gain insights into the interrelationship of ultradian rhythms and seizures. We focused on ultradian cycles in autonomic and activity data during a time window from 6 pm to 8 am, emphasizing nocturnal cycles. We identified seizure-related differences in ultradian cycles in heart rate, electrodermal activity and accelerometry recordings. Longitudinally collected data are required for determining causative inferences, i.e. whether changes in seizure risk mediate or are mediated by variations in the expressions of URs.

### Seizure occurrence relates to differences in ultradian rhythms

Body functions are organized by rhythmic patterns, which are often referred to as internal ‘clocks’.^44^ The related patterns contain health information and are disrupted in many diseases, among them epilepsy.^45^ In this study, we focused on the relationships between seizure occurrence and ultradian rhythms. However, beyond individual seizures, the neural mechanisms that underlie an individual’s enduring seizure propensity may themselves be associated with changes to such clocks. The occurrence of seizures at specific times of the day is a relevant characteristic of epilepsy.^44^ Ongoing efforts focus on detecting underlying patterns of this rhythmicity. Besides circadian and multiday cycles, shorter cycles might play a role. Those patterns are not present or well developed until after birth and they are gradually developed by a progressive maturation during infancy.^46,47^ To explore how seizure occurrence and heightened seizure risk may be associated with variations in ultradian rhythms, we compared wearable-derived physiological parameters between patients with epilepsy who either did (TCS) and did not (controls) experience a definite TCS during their EMU stay. Compared with controls, TCS patients displayed a relatively exaggerated 2-hour cycle in activity/accelerometry. TCS patients also displayed a significant lengthening of ∼4-hour long cycles in heart rate, while also displaying higher power across a range of ultradian frequencies in measures of electrodermal activity. At this stage, these findings remain largely correlative and provide limited evidence of directionality. Changes in ultradian phenotypes may be downstream of neurobiological processes that mediate high impending seizure risk or may serve a more upstream role by directly modulating these same processes. Ultradian oscillations may also be directly perturbed by seizure recency.

Generalized and focal onset seizures display unique patterns of chronobiology. While seizures of generalized onset often occur in the minutes/hours after morning awakenings, focal onset seizures (especially of frontal lobe origin) frequently emerge out of nocturnal non-rapid eye movement sleep. In line with this observation, during our recording time, which was selected based on data availability, the dataset mostly contains focal to bilateral TCSs. Overall, we found that most seizures occurred during the peak phase of heart rate rhythms, and trough phase of accelerometry rhythms. These results are preliminary and deserve large scale replication efforts to better reveal the directionality of these relationships, and their associations with abnormalities in circadian structure in patients with epilepsy.^48^ Ultimately, ultra-long recordings will be necessary to understand the relationships between ultradian, circadian and infradian rhythms in physiological variables that may correlate with seizure risk.^19^

A deeper understanding of the relationships between seizure occurrence and our many internal clocks provides several practical therapeutic implications. Beyond personalizing the timing of ASM administrations, identifying patient-specific relationships between seizure timing and ultradian phase/period length could refine the precision of existing seizure prediction/forecasting algorithms. As a corollary, avenues to reset or reshape ultradian rhythms (through physical activities, focused attention and relaxation tasks, or improved alignment of circadian and sleep/wake cycles) may provide antiseizure benefits without the classical side effects of ASMs.

### Limitations

Our study features a relatively small sample size and may have been influenced by selection bias and clinical confounders. Age-dependent changes in the expression of physiological ultradian rhythms have not been studied systematically. The current matching approach allowed us to account for the age and sex of the patients. However, the inclusion criteria allowed TCS patients to have additional non-TCSs, while controls remained seizure-free. Extending the results to other seizure types would require a larger dataset. Given the lack of a healthy control group, our results do not shed light on epilepsy-specific alterations in ultradian rhythms. Due to limitations in data availability, we maximized the temporal alignment of data with wearable recordings captured between 6 pm and 8 am. Our recordings were also conducted within the EMU, where the benefits of gold-standard EEG recordings must be weighed against the confounds of a non-habitual hospital environment. Nevertheless, our initial results provide a valuable baseline for future longitudinal ambulatory analyses of ultradian phenotypes in individual patients.

### Potential covariates and confounding determinants of ultradian rhythms

Precise details regarding the role of age-, sex- and other demographic determinants of ultradian rhythms in healthy control populations remain largely unknown. Further, the durability or inertia of ultradian rhythms has also been poorly explored, i.e. whether ultradian clocks at all reset when shifting from the native (home) environment to the EMU. Furthermore, individual schedules such as meal, school, and bed timings as well as social interactions may also have an impact on ultradian and circadian rhythms and at times align these patterns. Additionally, the disruption of those patterns, potentially also by seizures, impacts the internal clock and therefore alters ultradian patterns. In our cohort, the stay at the EMU might have disrupted normal interaction patterns and zeitgeber. Another covariate, which relates to age as well as the EMU setting, is hormonal changes, including long-term hormone adjustments, e.g. due to development and puberty, but also short-term, e.g. due to stress. Other conditions that impact the cyclic activity of the ANS, such as medical and psychiatric comorbidities, and/or prescribed medications might be additional confounders. Specifically, the type of ASM, ASM dosing and blood levels may play a role. Previous data suggest that heart rate variability is lower during recordings with high ASM doses.^49^ Additionally, the medication intake times will affect when and how strongly the ASMs influence the ANS activity. Some medications are taken daily, mostly in the evening, while others are taken every 12 or 8 hours. Many patients receive multiple ASMs, with potentially different intake times or intake cycles, hence making an estimate of ASM effects more challenging, and likely impacting cyclic activity within the ANS, and effects may also change based on age. Further, the interaction of sleep and wakefulness may be disjointed from circadian cycles, in particular in patients in an inpatient setting. The complex interplay of developmental stage, epilepsy, and resulting seizures, recorded in a specific environment, such as the EMU, with ASMs and their impact on wearable data might be key to epilepsy treatment. This study is underpowered to evaluate how ASM intake (type, dose and timing of treatment) may be associated with variations in ultradian rhythm expression and was not designed to separate the role of fluctuating seizure risk and fluctuating serum ASM levels in relationship to ultradian rhythms.

### Generalizability

This exploratory study has limited generalizability. Our aim was to obtain initial insights to generate a hypothesis for testing that may subsequently explore generalizability.

## Conclusion

Our data support the possibility that seizures and/or the dynamic fluctuations in seizure risk may be associated with alterations in the expressions of ultradian rhythms, as assessed by an unobtrusive wrist-worn wearable device. Larger studies are necessary to expand these observations to include patients with non-motor seizures and pure electrographic seizures, and to examine whether strategies to modify the phase and amplitude of specific rhythms may impart antiseizure benefit. In addition, we value this approach as an avenue to improved epilepsy management and seizure monitoring, especially in children with developing circadian and multiday rhythms.

## Supplementary material

Supplementary material is available at *Brain Communications* online.

## Supplementary Material

fcae034_Supplementary_Data

## Data Availability

All statistical analyses and results are included in the manuscript. Codes are not shared, but the packages used are listed and a link is provided in the manuscript. The original data are available upon reasonable request and when compatible with the BCH IRB requirements.
