# Supplementary material for: Ultradian rhythms in accelerometric and autonomic data vary based on seizure occurrence in paediatric epilepsy patients
Source: Brain Commun. 2024 Feb 12;6(2):fcae034. doi: 10.1093/braincomms/fcae034 (PMC10919479; doi:10.1093/braincomms/fcae034)
Supplement: fcae034_Supplementary_Data [file fcae034_supplementary_data.pdf]

Supplementary Table 1. Other seizure semiologies for the 29 patients with tonic-clonic seizures. Nine patients had 21 non-tonic-clonic seizures. Abbreviation: ILAE, International League Against Epilepsy. *N represents the number of patients; some patients are represented in more than one group. **n represents the number of seizures.

Timeframe	Total		Before 6 pm		From 6 pm – 8 am		After 8 am	
ILAE 2017 seizure semiology	N*	n**	N	n	N	n	N	n
Focal onset	7	14	1	2	5	10	2	2
Impaired awareness	5	6	0	0	3	4	2	2
Motor	4	5	0	0	3	4	1	1
Automatisms	1	1	0	0	0	0	1	1
Clonic	2	2	0	0	2	2	0	0
Hyperkinetic	1	1	0	0	1	1	0	0
Tonic	1	1	0	0	1	1	0	0
Nonmotor	1	1	0	0	0	0	1	1
Behavior arrest	1	1	0	0	0	0	1	1
Off camera	0	0	0	0	1	1	0	0
Generalized onset	2	7	0	0	1	1	2	6
Motor	1	1	0	0	0	0	1	1
Tonic	1	1	0	0	0	0	1	1
Nonmotor	1	6	0	0	1	1	1	5
Typical	1	6	0	0	1	1	1	5

Supplementary Table 2. Types of anti-seizure medication (ASM) per group.

Demographics variable	Answer categories	Reported in	All patients	Seizure group	Control group
Type of ASM**	None	N	8	2	6
	Brivaracetam	N	2	2	0
	Clobazam	N	17	10	7
	Clonazepam	N	1	1	0
	Clorazepate	N	1	1	0
	Diazepam	N	1	0	1
	Eslicarbazepine acetate	N	1	1	0
	Ethosuximide	N	2	1	1
	Felbamate	N	2	0	2
	Gabapentin	N	1	1	0
	Lacosamide	N	13	8	5
	Lamotrigine	N	13	8	5
	Levetiracetam	N	13	6	7
	Lorazepam	N	9	7	2
	Midazolam	N	1	0	1
	Oxcarbazepine	N	13	8	5
	Phenytoin	N	1	0	1
	Pyridoxine	N	3	1	2
	Rufinamide	N	1	1	0
	Topiramate	N	2	2	0
	Valproic acid	N	11	4	7
	Zonisamide	N	8	4	4
